# Automatic Resting Tremor Assessment in Parkinson’s Disease Using Smartwatches and Multitask Convolutional Neural Networks

**DOI:** 10.3390/s21010291

**Published:** 2021-01-04

**Authors:** Luis Sigcha, Ignacio Pavón, Nélson Costa, Susana Costa, Miguel Gago, Pedro Arezes, Juan Manuel López, Guillermo De Arcas

**Affiliations:** 1 Instrumentation and Applied Acoustics Research Group (I2A2), ETSI Industriales, Universidad Politécnica de Madrid, Campus Sur UPM, Ctra. Valencia, Km 7, 28031 Madrid, Spain; luisfrancisco.sigcha@upm.es (L.S.); juanmanuel.lopez@upm.es (J.M.L.); g.dearcas@upm.es (G.D.A.); 2 ALGORITMI Research Center, School of Engineering, University of Minho, 4800-058 Guimarães, Portugal; ncosta@dps.uminho.pt (N.C.); susana.costa@dps.uminho.pt (S.C.); parezes@dps.uminho.pt (P.A.); 3 Life and Health Sciences Research Institute (ICVS), School of Medicine, University of Minho, 4710-057 Braga, Portugal; miguelgago@hospitaldeguimares.min-saude.pt

**Keywords:** machine learning, wearable sensors, resting tremor, deep learning, convolutional neural networks, Parkinson’s disease, multitask

## Abstract

Resting tremor in Parkinson’s disease (PD) is one of the most distinctive motor symptoms. Appropriate symptom monitoring can help to improve management and medical treatments and improve the patients’ quality of life. Currently, tremor is evaluated by physical examinations during clinical appointments; however, this method could be subjective and does not represent the full spectrum of the symptom in the patients’ daily lives. In recent years, sensor-based systems have been used to obtain objective information about the disease. However, most of these systems require the use of multiple devices, which makes it difficult to use them in an ambulatory setting. This paper presents a novel approach to evaluate the amplitude and constancy of resting tremor using triaxial accelerometers from consumer smartwatches and multitask classification models. These approaches are used to develop a system for an automated and accurate symptom assessment without interfering with the patients’ daily lives. Results show a high agreement between the amplitude and constancy measurements obtained from the smartwatch in comparison with those obtained in a clinical assessment. This indicates that consumer smartwatches in combination with multitask convolutional neural networks are suitable for providing accurate and relevant information about tremor in patients in the early stages of the disease, which can contribute to the improvement of PD clinical evaluation, early detection of the disease, and continuous monitoring.

## 1. Introduction

Parkinson’s disease (PD) is a neurodegenerative disease associated with progressive dopaminergic nigro-striatal dysfunction, one of the main neural networks responsible by coordinating human movements [1,2]. Worldwide, an estimated 7–10 million people are living with this disease, and its prevalence increases with age, being rare before age 50 and more common in men than in women [3,4,5,6]. The prevalence of PD increases with age and PD affects 1% of the population above 60 years of age [7].

Several symptoms are present in PD. The most common symptoms are stiffness of the trunk and the extremities (increased muscle tone), slowness of movement (bradykinesia), rigidity, tremor (resting tremor and re-emerging postural tremor), postural instability, and gait impairment [8,9]. Among these symptoms, resting tremor is usually the most evident and clinically distinctive [10].

Specific medications such as levodopa and dopamine agonists remain the most effective drugs, at least in early clinical phases [11]. However, after several years of treatment, these therapies decrease their effectiveness and produce side effects such as motor fluctuations and dyskinesias [12,13].

Tremor is the involuntary oscillatory and rhythmic movement produced by synchronous or alternating contractions of agonist/antagonistic muscles. Tremor can be experienced in the hands, head, trunk, or legs [14]. In PD, tremors can appear in the early stages of the disease and reduce the quality of life by interrupting activities such as reading, writing, and eating [15]. More than 70% of all PD patients experience resting tremors in the course of the disease and their effects tend to be more severe with aging [16].

Tremor in PD can be divided into resting tremor, which occurs when patients relax their muscles, and action tremor (postural and kinetic) which occurs while the subjects make voluntary muscle movements [17]. The tremor usually occurs at a frequency of between 3.5 and 7.5 Hz [18], although different frequency ranges can be found in the related literature such as 3–5 Hz [19] or 4–6 Hz [20].

The current standard for PD evaluation consists of a clinical examination of patients by a neurology specialist, usually in an ambulatory hospital clinical setting and in sparse visits per year. In these examinations, medication scheme and dosing are adjusted, based on self-reported symptoms and a brief assessment of motor function. Although this method is widely used, the results depend on subjective clinical judgment and the patient’s report, potentially compromised by wrong self-perception due to cognitive impairment, making it difficult to accurately monitor the patient’s condition and disease progression [21]. Therefore, there is a need for continuous and objective monitoring of motor symptoms in PD to improve the therapeutic regimen and enhance the outcomes of clinical trials [22,23].

In the literature, several works have analyzed PD tremors using sensor technologies such as electromyography (EMG) [24,25,26], electromagnetic motion trackers [27], or noncontact measurements obtained from devices such as Kinect [28] or laser Doppler vibrometers [29]. However, the use of accelerometers or gyroscopes has been of particular interest due to their compact size, which allows their integration into portable systems [22,30].

The use of smart technologies for PD applications has increased in recent years, being important complementary clinical tools in early diagnosis and objective quantification of symptoms over time [30]. Data collected through wearable technology, combined with the capabilities of artificial intelligence to analyze data employing machine learning algorithms, can be used to estimate the severity of the tremor with high accuracy, based on the analysis of movement patterns obtained from different sensors.

Early studies have used gyroscopes [15,18,31] to detect tremor and other PD symptoms, as well as accelerometers for clinical and ambulatory monitoring [32,33,34,35]. Some approaches have proposed the use of several sensors placed on different parts of the body [32,33,35,36,37,38,39], while recent approaches tend to use sensors placed on the wrist, fingers [40,41,42,43], foot [44], and smartphones with writs adapters or watch-like devices [45,46,47,48,49,50]. Also, these approaches have been used in companion with automatic classification algorithms [36,37,38,39,40,46], threshold approaches [51,52,53,54,55], and deep learning technics [15,49,50,56] to provide low cost and non-invasive solutions for remote monitoring [45,57].

Although the use of wearable technology shows high potential as a complementary tool for clinical assessment, challenges remain in the development of systems and algorithms for automated monitoring of PD symptoms. For example, there is a need to develop autonomous monitoring systems capable of analyzing symptoms with high confidence to reduce subjectivity in the assessment and provide relevant information for the clinical assessment.

Additionally, affordable systems must be developed to improve the monitoring protocol through continuous tracking of the symptoms over time, taking advantage of the processing and communication capabilities of smart technology. Furthermore, the development of systems based on wearable devices that can be used in an unobtrusive fashion (such as watches or wristbands) [22] without increasing the burden to the patients due to the use of specific sensorization or the use of several sensors placed on the body is required.

Hence, this paper investigates the feasibility of using nonmodified consumer smartwatches and deep learning techniques to provide a self-contained and low-cost alternative for automatic detection and assessment of the constancy and amplitude of the resting tremor in patients with PD. This may allow improvements in the results of clinical trials through continuous and unobtrusive monitoring.

In this work, the use of a hierarchical approach developed to operate with deep learning models that analyze multiple tasks simultaneously (context and symptom) is proposed. The use of multitask models can help to improve the model’s generalization by leveraging the domain-specific information contained in the training data of related tasks [58]. To the best of our knowledge, this is the first study that proposes the use of a pipeline for simultaneous task analysis that takes advantage of multitask convolutional models for the evaluation of resting tremors.

For the development of this system, the frequency response of the embedded accelerometer has been analyzed using an adapted version of the calibration method for vibration transducers by comparison with a reference accelerometer. This analysis was made to identify the accuracy of a consumer smartwatch to measure the amplitude of the tremor, to be used as a robust indicator of clinical severity.

A data collection protocol has been developed for the evaluation of resting tremors. With the data collected for the smartwatches, different machine learning classifiers and data representations proposed in the related literature have been evaluated for the detection of resting times (context classifier) and the detection of the occurrence of tremors in the upper limbs (tremor detector). Additionally, novel approaches based on multitask convolutional neural networks (multitask CNNs) capable of simultaneously analyzing the context and the occurrence of tremors have been developed and tested.

The vast majority of experiments to evaluate the classifiers have been made using the leave-one-subject-out (LOSO) cross-validation methodology to identify the ability of the evaluated models to generalize data from unseen patients.

This study was carried out using data from PD patients who participated in the TECAPARK project [59]. These data include weekly records of 18 PD patients who manifested motor symptoms while performing a variety of scripted activities, including standardized exercises and upper limb resting periods. The present approach has been compared to the clinical assessment performed according to the ordinal Unified Parkinson’s Disease Rating Scale (UPDRS) [60] parts 3.17 (rest tremor amplitude) and 3.18 (constancy of rest tremor), to validate the results and identify reliable biomarkers for resting tremor monitoring that can support clinical evaluation, disease monitoring, and decision making.

The remainder of this paper is organized as follows: Section 2 presents the background, including the related work regarding the tremor assessment. Section 3 presents the materials and methods proposed in this work, including the data collection and evaluation methodologies proposed for tremor assessment based on multitasking analysis. Section 4 presents the experiments and results obtained from the evaluation of the proposed system and the methodology for tremor assessment. Finally, Section 5 presents the discussion and the conclusions of the results obtained in this work.

## 2. Background

Currently, the severity of resting and action tremor is analyzed during routine clinical visits using Part III of the UPDRS scale. During the assessment, patients were asked to perform three tasks: armrest, arm extension, and nose tip contact with the index finger (finger to nose test) [60].

While the patient performs these tasks, the maximum amplitude produced by the tremors was analyzed and rated on a scale ranging from 0 to 4 (where 0 implies that there is no presence of tremors and 4 indicates tremors with an amplitude of up to 10 cm).

Furthermore, as part of the UPDRS at the end of the evaluation, the constancy of the tremor was evaluated considering the percentage of time that a patient presented tremors during his/her entire examination. In a similar way to the other sections of the UPDRS, the constancy is qualified on a discrete scale from 0 to 4. Although this type of evaluation is a widespread method, visits to the specialist are spaced several months apart and often fail to capture the full spectrum of symptoms that the patients with PD may experience in their daily lives [61]. Therefore, tools for remote monitoring could help to improve treatments by collecting data in-home settings, reducing the number of clinic visits in situations similar to those produced by the COVID-19 pandemic emergency, where medical appointments have experienced a significant reduction.

In the literature, several works have analyzed the use of wearable devices in health care applications [62,63,64]. Many studies are focused on the development and validation of systems for the analysis and quantification of motor symptoms remotely using a variety of inertial sensors. The most commonly used sensors include accelerometers and gyroscopes due to their compact size and affordability [65,66,67,68,69,70].

The sensors named micro-electromechanical (MEMS), due to their low cost, have contributed to the development of compact monitoring systems, which can be used for monitoring PD and other movement disorders continuously [71]. Additionally, systems employing machine learning techniques have allowed the improvement and automation of PD motor symptom detection with higher speed and reliability than standard analysis methods [65,67,68]. As a result, these technologies have led to the development of solutions for automated and continuous monitoring, allowing a reduction in overall cost [72]. 

An important aspect considered in the related literature has been the quality of the data obtained from this type of device and sensors because it has a significant impact on the performance of these systems. Thus, an appropriate selection of sensors and algorithms have supported the improvement in the performance of these systems, also considering the computational costs for real-time applications, such as those related to health care, where a trade-off between performance and efficiency is required [73].

The approaches identified in the literature have reported high performances in identifying tremors and high correlations with UPDRS scores. The main differences found in these studies are related to the configuration (single device or multimodal devices), the number of sensors, the positioning of these sensors in the patient’s body, and the monitoring protocol such as temporary evaluation or continuous evaluation. Table 1 summarizes previous highlighted works since 2010, regarding the development and validation of tremor analysis systems using wearable sensors. 

Early studies by Salarian [18,31] have used spectral analysis from signals obtained from gyroscopes placed on the wrists to detect tremors, showing symptom detection with a specificity of 99.5% and sensitivities of up to 94.2%. Keijsers et al. [33] obtained specificities and sensitivities of 0.97 using six accelerometers placed on different parts of the body. Giuffrida et al. [34] have shown high correlations (r^2^ = 0.89) when comparing the root-mean-square (RMS) magnitude of accelerometers and gyroscopes with the clinical scores. 

In recent years, most of the works concerning the analysis of tremors have used supervised machine learning techniques to identify the presence of tremors using different types of “hand-crafted” features extracted from inertial signals in both time and frequency domains [15,36,37,38,39,40,46,55,56,57]. Other authors have used threshold-based algorithms, taking advantage of the heuristic knowledge of the symptom to determine objective indicators for monitoring the symptom [43,45,47,51,52,53,54,55].

Some other approaches [15,49,50,56] have started to use deep learning techniques for tremor detection, mainly Convolutional neural networks (CNNs) [74] and recurrent neural networks such as Long short-term memory (LSTM) [75], showing, in several experiments, a higher performance than the methods based on shallow algorithms. The deep learning techniques have enabled the development of end-to-end classifiers used in many applications such as human activity recognition (HAR), using data acquired from multiple sensors [76] or data from sensors of smart devices such as smartphones or smartwatches to detect the user context or the activity performed at any given time [77].

Among the solutions based on wearable technology, the use of consumer devices such as smartphones [45,57] and smartwatches [51,54] has been identified. These studies show that the use of smart devices is feasible, reliable, and well-related to clinical scores, whereas in the case of smartwatches they show good acceptance from the patients, which may allow their use as continuous monitoring devices.

Although a great advance can be identified in the area of PD tremor monitoring, several challenges have been identified that need to be addressed. For example, few studies have focused on characterizing, from a metrological point of view, the amplitude or the frequency response of the accelerometers of consumer devices to provide accurate indicators for clinical monitoring and assessment. Additionally, the results of the detection systems are not always associated with clinical ratings and in some cases lack the expected clinical outcomes for enhanced monitoring. Additionally, it is desirable to reduce the number of devices or sensors placed on different parts of the body to improve usability in free-living environments.

Finally, differences between our work and the reviewed studies can be categorized as follows:

- Several studies have evaluated systems for tremor detection without distinguishing the type of PD tremor (rest or action tremor); usually, these approaches are focused on free-living monitoring [15,37,38,39,43,45,47,52,53,56]. Instead of only measuring features related to PD, data analysis techniques should be used to identify reliable biomarkers to support the clinical assessment that can be obtained automatically with wearable technology and artificial intelligence techniques [68];
- Early studies have used configurations of several sensors placed on different parts of the body [15,36,37,38,39], but these systems could present usability limitations in ambulatory settings. In this work, the use of a nonmodified consumer smartwatch is proposed in contrast to the use of multimodal systems or ad-hoc devices for data acquisition;
- Several studies have used threshold or shallow machine learning approaches to detect the presence or the amplitude of tremor, while few studies have used deep learning techniques [15,49,50,56]. However, the use of multitask convolutional models has not been identified for resting tremor assessment.

In contrast to the reviewed works, our study examines the capability of a system based on a deep learning multitask approach combined with the data acquired from a single (triaxial) accelerometer sensor of a nonmodified consumer smartwatch for tremor assessment (amplitude and constancy). Additionally, the use of standardized metrics for measuring vibration amplitude such as the acceleration level is compared with the clinical rating to validate the results of the proposed system.

## 3. Materials and Methods

This section describes the materials and methods used in the data collection and evaluation of the proposed systems. This section has been divided into five subsections. Section 3.1 shows the data collection methodology and the characteristics of the group of patients involved in the experiments. Section 3.2 shows the analysis methodology and the frequency response obtained from the evaluation of the acquisition device. Section 3.3 shows the experimental protocol for data acquisition based on a smartwatch and a custom software application. Section 3.4 shows the methodology for data labeling. Section 3.5 describes the algorithmic approaches used to the development of different classifiers (context and tremor), as well as the proposed approach for the assessment of the amplitude and constancy of the resting tremor using models with multiple outputs.

### 3.1. Data Collection

Data were collected during the TECAPARK project using a custom-built mHealth mobile and wearable application for tracking motor symptoms of PD patients using smartphones and smartwatches [59]. The study was approved by the Ethics Committee of the Universidad Politécnica de Madrid. The PD patients were previously diagnosed according to the UK Parkinson’s Disease Society Brain Bank [78]. All subjects gave their written consent before participating in the experiment.

A total of 18 subjects with PD were recruited from different Parkinson associations located in Spain and Portugal who were in early stages (≤2) of the disease according to the Hoehn and Yahr (H&Y) scale [79] (age: 64.9 ± 7.6 [47,48,49,50,51,52,53,54,55,56,57,58,59,60,61,62,63,64,65,66,67,68,69,70,71,72,73,74,75,76] years; gender: 8 M/10 F; stage H&Y I/II/: 4/14). Patients had a good clinical response to levodopa and/or dopamine agonists and did not present dementia according to the diagnostic and statistical manual of mental disorders IV (DSM IV) criteria [78].

From this group of 18 patients, 4 patients did not present tremors, while the remaining 14 presented tremors with different severities. Amplitude tremor ranged from 0 (no tremor) to 2 (mild tremor) according to the UPDRS guide section 3.17. Tremor constancy ranged from 0 (no tremor) to 4 (tremor is present more than 75% of the entire examination period), evaluated according to the UPDRS guide section 3.18. Patients were assessed in their best ON state (the ON state is the one in which motor symptoms are controlled by the medication) as assessed by clinical and patient’s assessment history. During the study, all patients continued taking their medication as usual.

**Figure 1:**
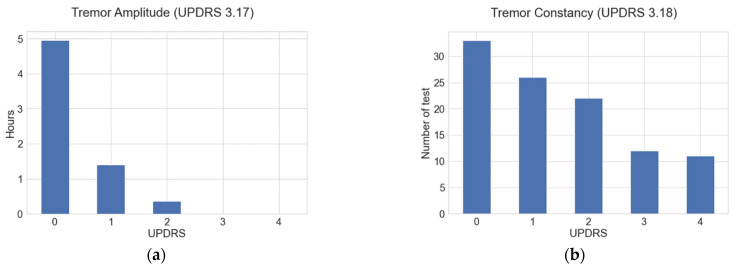
(**a**) Number of hours data were analyzed, distributed according to the tremor amplitude severities evaluated through the UPDRS guide section 3.17; (**b**) number of constancy tests distributed according to the UPDRS guide section 3.18.

Figure 1a shows the number of hours that resting tremor signals were analyzed for in this study; they are distributed according to the amplitude severity evaluated with UPDRS guide section 3.17, while Figure 1b shows the number of tests performed for the tremor constancy according to the UPDRS guide section 3.18.

The data generated in the experiments were stored in the internal memory of the smartwatch and downloaded later for labeling and off-line evaluation. The experiments for data processing and evaluation were conducted on a computer with an Intel Xeon with 2.30 GHz processor, 12 GB of random-access memory (RAM), and a 12 GB NVIDIA Tesla K80 graphics accelerator card. The signal labeling, preprocessing, and feature extraction were performed using the MATLAB software (version R2017a), while the evaluation and training of the classification models were performed in Python (version 3.6), using the libraries Keras (version 2.4) [80], TensorFlow (version 2.3) [81], and Scikit-learn (version 0.22) [82].

### 3.2. Acquisition Device (Smartwatch)

A consumer smartwatch was used for data acquisition; the device was placed on the wrist of the most affected side of each patient. The smartwatch was available on the market in 2019 and uses the Android Wear operating system. The device has an internal memory of 4 GB (2 GB of free space), the dimensions are 46.6 mm × 51.8 mm × 12.9 mm, and it has a weight of 32.5 g. The device has a calibrated accelerometer, with a maximum amplitude set to ±2 g. The sensor can be adjusted to a maximum sampling rate of 100 Hz factory-set by the operating system.

In this study, the sampling rate was adjusted to 50 Hz, which is considered a suitable value for human activity recognition (HAR) using sensors placed on the wrist (standard human activities do not typically exceed 10 Hz) [83,84] and is suitable for evaluating tremors in the 3.5–7.5 Hz range, as usually occurs in PD [18]. 

The smartwatch used for the data collection was previously analyzed to identify its frequency response by using a methodology described in a previous work [85]. The analysis employs an adapted version of the calibration method by comparison with a reference accelerometer. For this method, the smartwatch and a reference accelerometer, the Dytran 3023M3 (Dytran Instruments, Chatsworth, CA, USA), were simultaneously coupled to an electrodynamic vibration shaker, LDS V406 CE M4 (Bruel and Kjaer). The vibration signals were generated from a PULSE 7537 vibration analysis system (Bruel and Kjaer, Copenhagen, Denmark) and a power amplifier, LDS PA 100E (Bruel and Kjaer).

To evaluate the frequency response of the device, six root-mean-square (RMS) determinations were performed for different acceleration values at known frequencies and amplitudes using discrete sinusoidal signals.

**Figure 2:**
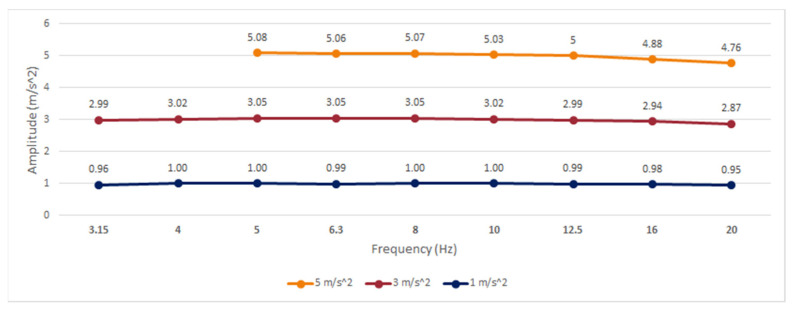
Frequency response of the smartwatch built-in accelerometer.

Three different amplitudes (1, 3, and 5 m/s^2^) were tested in this experiment for a single axis at standardized third-octave intervals, with central frequencies in the range of 3.15–20 Hz. Values for 3.15 and 4 Hz at 5 m/s^2^ could not be determined due to the limitations of the equipment to reach those magnitudes. The results of the frequency analysis at different amplitudes are shown in Figure 2, and the deviation percentage (ε) to the reference accelerometer is shown in Table 2.

According to the methodology established in [85], an error band has been considered for amplitude and frequency linearity according to the specifications of the International Organization for Standardization (ISO) 8041:2005 [86], in which values with a deviation greater than ±6% are considered linearity errors. 

For the smartwatch, the results show a linear behavior in frequency and amplitude that can be useful for the analysis of standard human activities and parkinsonian tremors, with amplitudes evaluated up to 5 m/s^2^. For frequencies higher than 16 Hz, a gradual decrease in amplitude was noted; however, the relative error did not exceed ±6% at any analyzed point, with a maximum error of −4.89% at 20 Hz. Expanded uncertainties equal to or less than ±3% were achieved in all experiments, following the ISO 8041:2005 recommendations for mechanical tests of amplitude linearity.

### 3.3. Experimental Protocol

The experimental protocol used for data acquisition was based on the combination of six scripted activities extracted from UPDRS, including some exercises to assess motor symptoms in PD, plus a resting time interval of at least 30 s between each exercise. We used UPDRS exercises because they are a well-known protocol for PD patients, therapists, and neurologists, and they simplify labeling the signals for comparison with clinical assessment.

**Figure 3:**
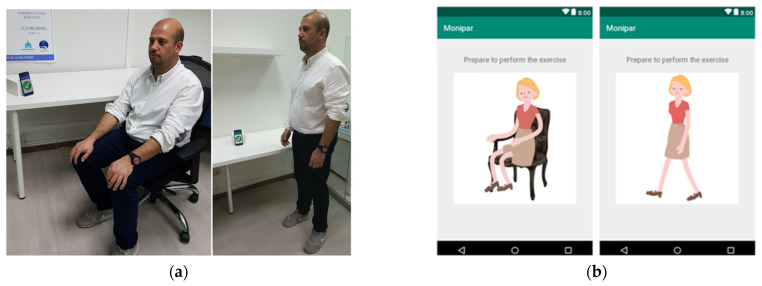
(**a**) Evaluation tests and data acquisition based on a smartwatch. (**b**) Mobile application.

To facilitate data collection, a custom mobile application was used to guide the patients to perform exercises using voice instructions as shown in Figure 3.

Patients were evaluated weekly in their care center with the supervision of the center’s staff during an interval of between 2 and 8 weeks (average 5.8 weeks). Complete evaluation, including exercises and resting times, took an average duration of 6 min per patient.

### 3.4. Data Labeling

Since the data labeling is a costly and time-consuming process, the data used were initially labeled using a method based on the analysis of the magnitude in the tremor band (3.5–7.5 Hz), and thresholds were empirically established to identify the presence of tremors. These labels were then reviewed and corrected by comparison with a video recording reference for each of the tests. The clinical evaluation of the tremor amplitude and constancy were performed by an expert according to the UPDRS guide sections 3.17 and 3.18.

For the context classifier, the resting periods were labeled by manually annotating the intervals from the video recordings.

### 3.5. Algorithmic Approach

A hierarchical approach has been developed for the evaluation of tremors in PD disease, analyzing in parallel the resting segments and the presence of tremors. This approach allows for the analysis of symptom markers such as the constancy and amplitude of the tremor.

In the proposed approach, the time segments in which the patient is at rest and does not make any movement with his hands are identified. From here onwards, this will be referred to as context classification. This classifier prevents the generation of false positives in the detection of resting tremors due to the performance of other types of activities. At the same time, a second classifier is used to detect the presence of resting tremors.

**Figure 4:**
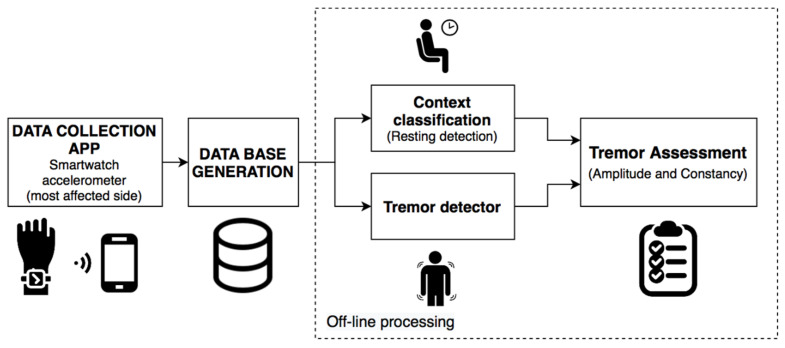
Algorithmic approach for the assessment of resting tremor.

For the assessment, in the segments in which the context classifier identifies that the patient is at rest and the tremor algorithm detects the presence of tremors, the symptom evaluation is carried out by analyzing the acceleration level in the tremor band. Additionally, the tremor constancy is analyzed by using the information of the resting and the tremor periods. The proposed approach was developed according to the scheme shown in Figure 4.

#### 3.5.1. Context Classifier

The context classifier is designed to automatically detect the time segments in which the patient is at rest, regardless of the presence of tremors. To validate the performance of the context classifier, the LOSO cross-validation was used with the data of all patients.

In the LOSO methodology, the data of all patients except one are used for the training of the model, the data of the remaining subject are used for the evaluation, and this process is repeated for each patient. The results obtained with the LOSO methodology are the average of the results of all patients.

To train and evaluate the algorithms, the signals of the triaxial accelerometer were divided into fixed intervals (windows) of 2.56 s (128 samples) with a 50% overlap which, according to [36], is a suitable time interval to analyze tremors with inertial sensors, while the increase in this time does not present significant improvements. A window was labeled as resting or nonresting only if more than 50% of its samples were labeled as resting or nonresting. Windows with samples containing unknown labels or windows with labels containing less than 50% of their samples labeled as a resting or nonresting were discarded during training.

The signals were filtered by a Butterworth band-pass filters with cut-off frequencies of 0.5 to 10 Hz and third-order slope, which is a suitable range for human activity recognition using sensors [84].

**Figure 5:**
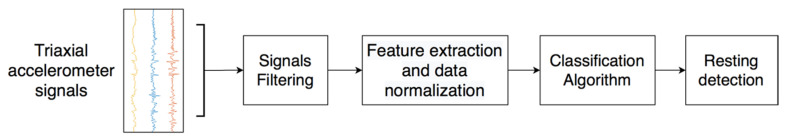
Algorithm for the context classifier using machine and deep learning techniques.

For the evaluation of the context classifier, several algorithms and three different types of data representations were tested. The context classifier was developed following the scheme shown in Figure 5, by using machine and deep learning techniques.

To create a baseline for context classifier, 290 hand-crafted features commonly used in context detection and automatic human activity recognition with smartphones and smartwatches were extracted from the acceleration signals [77]. These types of features are normally used to identify human physical activity (sitting, standing, walking, etc.); however, in this study they were used to detect the context of the patient corresponding to resting or nonresting periods.

The features extracted include time and frequency characteristics [77]. This data representation has been evaluated using an AdaBoost [87] classifier with 100 estimators. This algorithm was used as a baseline due to its good performance and because it does not require complex adjustment of hyperparameters for training.

To compare the results obtained with the baseline, a second data representation based on the frequency spectrum was implemented using the Fast Fourier Transform (FFT) [88] obtained after calculating the Euclidean Norm of the signals (Equation (1)). This spectral representation is used because in [40] the authors state that using characteristics extracted from the frequency domain can provide results with similar performance to those obtained by combining both time and frequency characteristics.

(1) $$a\left(i\right)=\sqrt{a_{x}^{2}\left(i\right)+a_{y}^{2}\left(i\right)+a_{z}^{2}\left(i\right)}$$

where $a_{x},\;a_{y},a_{z}$ are the acceleration values related to *x*, *y*, and *z* axes.

The FFT-based data representation was evaluated using AdaBoost and Gradient Boost classifiers [89].

As a second approach for the context classifier, a CNN was evaluated for classification with the raw signals as data representation, taking advantage of CNN’s capability to automatically extract discriminating characteristics from the signals [74]. For evaluating the CNN, the signals of the triaxial accelerometer were filtered in the interval from 0.5 to 10 Hz and normalized in a range from 0 to 1.

**Figure 6:**
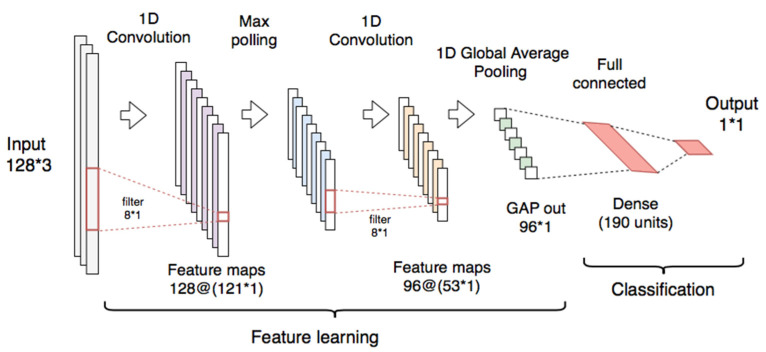
Convolutional neural network used for the context classifier using the raw signals.

The CNN used for the context classifier is composed of an input layer (128 samples and 3 channels) and two one-dimensional convolutional layers (1-D CNN) with Rectified Linear Unit (ReLU) activations. The first CNN layer has 128 filters (kernel = 8), and it is connected to a max polling layer (pool size = 2). The second CNN layer uses 96 filters (kernel = 8), followed by a global average pooling (GAP) layer [90] which calculates the average values of each of the feature-maps by reducing them to a dimension of 1 × 1. For the classification, a dense layer with 190 units (ReLU activation) was connected to an output layer with a sigmoid activation function to obtain the probabilities that the input samples correspond to a resting segment. The architecture used for the implementation of this classifier is shown in Figure 6.

Deep neural networks are difficult to train due to the large number of parameters that must be adjusted in each layer [91]. An accurate selection of these hyperparameters controls the training process and has a great influence on the performance of the model. The hyperparameter optimization was made by using the hyperband method [92]. During this process, the following hyperparameters were adjusted: learning rate, the number of CNNs and fully connected layers, the number of filters and kernel sizes in the CNN layers, the number of neurons in the fully connected layers, and the batch-size.

The training is an iterative process that repeats until finding an acceptable solution for a problem; in this process, the weights of the layers are updated iteratively. These iterations are named epochs. The correct adjustment of the number of epochs is important to prevent overfitting and unnecessary computing. The training of the CNN model was performed by the retro-propagation method using the Adaptive moment estimation (ADAM) [93] optimizer with a learning rate of 0.0046, the binary cross-entropy as loss function, a batch-size of 64, and 200 as the max number of epochs. To avoid overfitting, an early-stopping strategy was employed, which requires subdividing the training data into a proportion of 80% for training (train–train) and 20% for validation (train–validation). A reduced batch-size of 64 samples was chosen, allowing the model to achieve a better generalization capability [94].

#### 3.5.2. Tremor Detector

This detector was created to automatically detect the presence of tremors and was used in conjunction with the context classifier to assess the symptom. For the evaluation of this classifier, several feature extraction techniques and algorithms proposed in the literature were reproduced and evaluated using the signals of the triaxial accelerometer with windows of 2.56 s. A window was labeled as a tremor or nontremor only if more than 50% of its samples had these labels. Windows containing unknown labels or windows with labels containing less than 50% of its samples labeled as a tremor or nontremor were discarded during training.

For the development of the tremor detector, the scheme proposed in Figure 5 (context classifier) was used for machine and deep learning approaches.

The sets of features used to evaluate the tremor detector include: the spectral representation (FFT) obtained from the Euclidean Norm of triaxial signals (used as a baseline), features composed of the Mel frequency cepstral coefficients (MFCCs) adapted to inertial signals as used in [49,56], a set of features proposed by Hssayeni et al. [15], a set of features proposed by Mahadevan et al. [55], and the raw triaxial accelerometer signals used to evaluate the deep learning approaches.

As in the context classifier experiments, the signals were filtered in the range of 0.5–10 Hz, except for the Hssayeni et al. [15] data representation filtered in the range of 0.5–15 Hz. A summary of the data representations used to evaluate the tremor classifiers is shown in Table 3.

As in the context detector, a CNN model similar to that shown in Figure 6 was evaluated for tremor detection using the raw signals of the triaxial accelerometer as an input. The CNN was trained using the same hyperparameters of the context CNN and using the early-stopping strategy to avoid overfitting.

#### 3.5.3. Convolutional Multitask Models (Multitask CNN)

As a novel approach for the context classifier and tremor detector, a deep neural network with two independent outputs was implemented and evaluated. The proposed multitask CNN is capable of analyzing the context and the occurrence of tremors simultaneously. The model can reduce the number of trainable parameters that would be required when using two independent CNN models for context classification and tremor detection.

**Figure 7:**
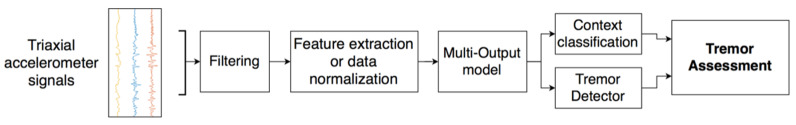
Algorithm for the tremor assessment using a multitask Convolutional neural network (CNN).

The scheme proposed for the tremor assessment using the multitask CNN models is shown in Figure 7.

The proposed model has convolutional and polling layers that are shared with two separated branches with independent fully connected layers used for classification.

The multitask CNN is composed of two 1-D CNNs (128 filters, kernel = 8, and ReLU activation). The first CNN is connected to a max polling layer (pool size = 2) and the second is connected to a GAP layer. The convolutional and the polling layers were used for feature learning. For classification, two individual branches composed of dense layers (each one with 190 units and ReLU activation and dropout of 0.2) were connected to single-output layers for context and tremor detection, each one with sigmoid activations.

**Figure 8:**
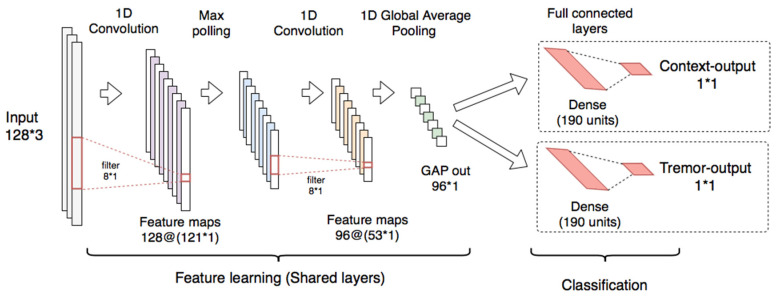
Multitask CNN used for the context classifier and the tremor classifier using raw signals.

The architecture used for the implementation of the multitask CNN classifiers is shown in Figure 8.

The multitask CNN was tested on the raw signals of the triaxial accelerometer and the FFT data representation. As in the context classifier and tremor detector, the triaxial signals was filtered in the range of 0.5 to 10 Hz and normalized in the range of 0–1. For the multitask CNN with FFT, the input layer was adapted to receive data of 64 features with a single channel (Input 64 × 1).

As in the CNN for context detection, the hyperparameter optimization was made by using the hyperband method to identify a suitable learning rate and the number of units in the fully connected layers of each branch.

The training of the model was performed by the retro-propagation method using an ADAM optimizer [93], with a learning rate of 0.0039 for the model with raw signals and 0.0046 for the model with FFT. The loss function selected to train the model was the binary cross-entropy. This loss function was used in the two branches with no weightings applied to the outputs. Additionally, a batch-size of 64 and 200 as the max number of epochs was selected. An early-stopping strategy was set in both models to avoid overfitting.

#### 3.5.4. Resting Tremor Assessment

For the assessment, both the amplitude and tremor constancy were evaluated using data of each experimental visit. These indicators were obtained to offer a long-term monitoring mechanism to improve the outcomes for the clinical evaluation of the disease.

**Figure 9:**
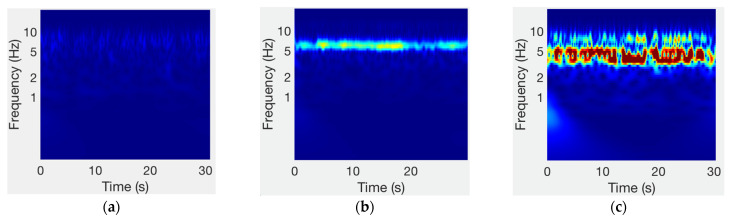
Spectrum of accelerometer signals for different tremor severities: (**a**) no tremor (UPDRS = 0); (**b**) slight tremor (UPDRS = 1); (**c**) mild tremor (UPDRS = 2).

The evaluation of the amplitude of the tremors was made through the spectral analysis of the inertial signals. In agreement with the literature, the spectral analysis on the accelerometer signals shows an increase in the level in the frequency band from 3.5 to 7.5 Hz [18] when the patient presents resting tremors (Figure 9).

In this study, to provide an amplitude indicator the acceleration level (La) of the RMS amplitude in the tremor band (3.5–7.5 Hz) was calculated for each analysis window. In windows in which the context detector detects that the patient is at rest and the tremor detector identifies the presence of tremors, the acceleration level was calculated according to Equation (2), using the reference acceleration as defined in ISO 1683 [95].

(2) $$La=20\log\frac{a}{a_{0}},$$

where a is the RMS acceleration (m/s^2^) and $a_{0}$ is the reference acceleration of 1 μm/s^2^.

The RMS values in the tremor band can be calculated from the frequency spectrum by using Pacerval’s theorem (Equation (3)).

(3) $$RMS=\sqrt{2\overset{f=SR/2}{\underset{f=0}{\sum }}{\left|X\left(f\right)\right|}^{2}},$$

where |*X*(*f*)| is the module of the FFT components in each spectral line.

After obtaining the acceleration level from all resting tremor windows, the 75th percentile was obtained to simulate the clinical analysis situation using the UPDRS scale in which the indicator of the amplitude of the tremors is the highest amplitude identified during the evaluation.

Following the methodology proposed in [55] to analyze the amplitude of the tremors, in this study it has been identified that using the acceleration level as a magnitude indicator, the 75th percentile presents a better agreement with the clinical evaluation through the UPDRS section 3.17.

For the evaluation of the tremor constancy, data from a weekly visit (6 min) were analyzed (see Section 3.3). To provide a constancy indicator, the segments in which the patient were at rest were compared with the segments detected as tremors, to obtain a percentage of time in which the tremor appears during the examination period. The results of the constancy of resting tremor were presented as a percentage to improve the resolution of the analysis, instead of showing them with a discrete scale (from 0 to 4) as it is usually made according to the UPDRS section 3.18.

## 4. Experiments and Results

For evaluating the proposed system, the performance of the context classifiers and tremor detection were analyzed separately. Once the best classifiers were identified, predictions were made with the whole system and their performances were evaluated by comparing them with the clinical evaluation made by a neurologist according to the UPDRS guidelines for tremor constancy and amplitude.

The classifiers were evaluated using the LOSO methodology. The complete LOSO process was repeated six times in each model to verify the variability in the results due to the stochastic processes in the training procedure. The LOSO methodology is more appropriate for the evaluation of data representations that use sliding-windows with overlap, to prevent signal segments to be shared between training and validation subsets.

This section has been divided into four subsections: Section 4.1 shows the results of the training of the multitask models. Section 4.2 shows the results of the context classifier. Section 4.3 shows the results of the tremor detector. Section 4.4 shows a comparison of the performance of the multitask CNN and the best models reproduced from previous works. Section 4.5 shows the results of the multitask CNN per patient. Section 4.6 shows a comparison of the performance of the deep learning models. Section 4.7 shows the results of the resting tremor assessment using the multitask models.

### 4.1. Results of the Training of the Multitask Models

**Figure 10:**
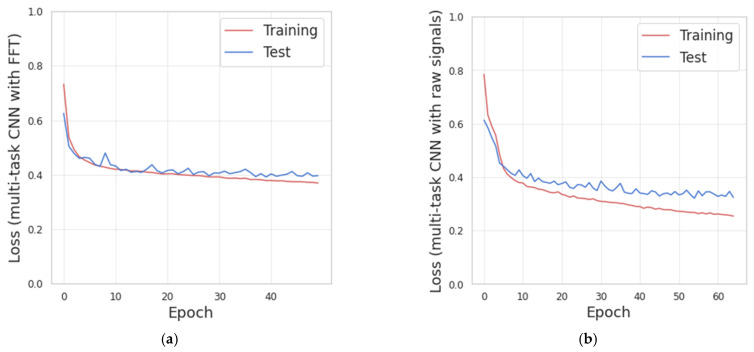
(**a**) Loss curves obtained from the training of multitask CNN with Fast Fourier Transform (FFT). (**b**) Loss curves obtained from the training of the multitask CNN with triaxial signals.

The convolutional multitask models shown in Section 3.5.3 have been implemented and trained with the data representations based on the FFT and the triaxial accelerometer signals. The resulting loss curves for the training and test subsets of two models trained during LOSO evaluation are shown in Figure 10.

Figure 10 shows that increasing the number of training-iterations (epochs), the value of the losses decreases for the training and test subsets. The early-stopping strategy was implemented with the test-loss as monitor and the patience parameter set to 10.

Figure 10a, corresponding to the multitasking CNN with FFT, indicates that there is no significant improvement in the test-loss after epoch 62. Thus, overall, 72 epochs were performed. The mean processing time for this model was 4.6 ms per batch (batch size of 64 samples) and 1.9 s per epoch.

Figure 10b, corresponding to the multitask CNN with triaxial signals, indicates that there is no significant improvement in the test-loss after epoch 55. Thus, overall, 65 epochs were performed. The mean processing time was 6 milliseconds per batch and 2.4 s per epoch.

These results indicate that the multitask models were properly trained with the configuration indicated in Section 3.5.3.

The mean processing time in the multitask CNN with a raw signal was slightly higher than the model with FFT. This increase is because the model needs to handle a larger amount of data corresponding to the three channels from the accelerometer and the model needs to adjust a greater number of parameters in the training.

Additionally, the presence of overfitting was not identified because of the loss curves for validation and testing decrease until the training was stopped by the early-stopping strategy. The early-stopping was configured to restore the weights from the epoch with the lower test-loss.

### 4.2. Results of the Context Classifier

For a binary classification problem (resting and nonresting) such as this, the results obtained from the context classifier are expressed by sensitivity, specificity [96], and the area under the curve (AUC) of the receiver operating characteristic curve [97]. The sensitivity is the ratio of positives that are correctly identified, while specificity is the ratio of negatives that are correctly identified. The values of sensitivity and specificity have been obtained using a threshold of equal sensitivity and specificity.

Three data representations have been used for the evaluation of this classifier and several classification algorithms have been evaluated such as AdaBoost (100 estimators), Gradient Boost (100 estimators), a CNN model trained with triaxial raw signals (128 samples × 3 channels) as indicated in Section 3.5.1, and two CNN multiout models trained with raw signals and FFT, respectively (see Section 3.5.3). The results achieved for the context classifier and its standard deviations (in parentheses) are summarized in Table 4.

The results obtained for the context classifier show a similar performance between the baseline (HAR features with AdaBoost) and the spectral representation (FFT) with the AdaBoost, with sensitivities reaching 88.6%. A slight increase in sensitivity and specificity was achieved when using a Gradient Boost classifier with the spectral representation; additionally, similar results were achieved using the proposed CNN (single output) with the raw signal as data representation, reaching a sensitivity and specificity of 89.8%, and AUC up to 0.962. The best results were achieved using the CNN Multiout models trained with both raw signals and the FFT.

### 4.3. Results of the Tremor Detector

The results for the tremor detector were achieved using the LOSO methodology. For the LOSO evaluation, the results are the average of the partial results from the 14 patients who presented tremors.

Several systems and algorithms have been evaluated including Support Vector Machines (SVMs) [98] (with a radial basis function kernel, gamma equal to 0.3, and penalty parameter equal to 1), AdaBoost (100 estimators, with FTT and MFCCs), Random Forest [99] (with 10 estimators as stated in [55]), Gradient Boost (with 70 estimators as stated in [15]), a CNN (single output) with raw signals, and two multitask CNN models trained with raw signals and the FFT features.

For the tremor detector, the SVM classifier with the FFT data representation was used as a baseline. The results obtained in these experiments and their standard deviations using LOSO evaluation are shown in Table 5.

According to the results shown in Table 5, the two multitask CNN models present higher performances in sensitivity and specificity; however, the system proposed by Hssayeni [15] is the best shallow machine learning approach and presents a higher AUC value than the multitask CNN with raw signals but presents similar results in terms of sensibility and sensitivity (84.1% and 84.0%, respectively). According to these results, the performance of this system could be considered for the implementation of a system based on independent classifiers with shallow machine learning algorithms.

The AdaBoost algorithm with FFT also presents a good performance over the CNN (single output), but less than the multioutput models. The remaining approaches have shown AUC results up to 0.9, with sensitivities and specificities below 84%.

The best result in tremor detection has been achieved using the multitask CNN with the FFT.

### 4.4. Comparison of the Multitask CNN Models with Previous Works

In this section, the best results obtained in the context classifier and the tremor detector are analyzed. Additionally, for comparison purposes, the results obtained for both classifiers are reported with the LOSO evaluation and the stratified 10-fold cross-validation.

For the stratified 10-fold evaluation the dataset was divided into 10 folds that were created by preserving the percentage of samples for each class. From these folds, nine were used for training and one for testing, and the process was repeated for each fold. The results of the evaluation are the mean of the ten iterations. The use of the stratified 10-fold cross-validation could lead to achieving over-optimistic results since the training and test data are usually very similar.

Table 6 shows a summary of the best results obtained from the systems reproduced from previous works and the results achieved with the multitask CNN models for both context and tremor classification. The results are presented in terms of AUC for the LOSO and the stratified 10-fold cross-validation.

According to Table 6, the proposed models based on multitask CNN models show a slight improvement in terms of AUC over the best results obtained from the reproduction of previous works in both classification tasks and evaluation methodologies. As expected, the results obtained from the stratified 10-fold cross-validation presents higher performances over the LOSO evaluation.

The use of spectral representations seems to be suitable for accurate tremor detection, while the use of complex data representations (including time and frequency features or raw signals) seems to be suitable for context identification.

The results obtained in the LOSO evaluation present a higher standard deviation than the stratified 10-fold validation. These variations are produced because some patients present different movement and tremor patterns, thus producing a higher variation in the global results.

In contrast, the stratified 10-fold evaluation presents a low standard deviation regardless of the system or the classification task, because of the similarity between the training and test subsets.

A further comparison for each classifier (context and tremor) is provided in Section 4.4.1 and Section 4.4.2.

#### 4.4.1. Comparison with Previous Works for the Context Classifier

For the context classifier (resting periods), our best results were obtained with the multitask CNN with the raw triaxial signals from the accelerometer. The best context classifier shows a sensitivity of 92.0%, a specificity of 93%, and an AUC of 0.981, evaluated through LOSO cross-validation. This classifier shows an increase in the performance over the baseline (time and frequency features used in HAR [77] with AdaBoost) up to 4.4% in the sensitivity and specificity.

In terms of AUC, an increase of 0.02 was achieved over the baseline in both evaluation methodologies. For the context data, a difference of 0.01 in AUC is significant with *p*-value < 0.001, according to Hanley’s method [97].

Additionally, a slight improvement in the performance over the baseline has been shown in Table 4 using different data representations such as FFT and a Gradient Boost classifier. While the CNN (single output) with raw signals present similar results to the best shallow machine learning approach (FFT with Gradient Boost).

In addition to the improvement in the performance of the context classifier when using models with multiple outputs, it can be observed according to Table 4 and Table 5 that the performance of the tremor detector is also increased. This improvement could be produced because the outputs of the CNN multioutput models share convolutional layers that are used to automatically learn discriminating characteristics by the two branches of the classifier, thus increasing the generalization capacity of the network and allowing the convolutional layers to learn discriminant characteristics useful for both context classification and tremor detection.

#### 4.4.2. Comparison with Previous Works for the Tremor Detector

The best result obtained in the present study for detecting resting tremors shows a sensitivity of 86.1%, a specificity of 86.1%, and an AUC of 0.936 using a multitask CNN with FFT, evaluated through LOSO evaluation. These results show an increase in the performance—5% increase in sensitivity and 5.6% for the specificity when compared with the baseline (SVM with FFT), and 2% (sensitivity and specificity) higher than the best system [15] reproduced from previous works.

In terms of AUC, an increase of 0.014 and 0.009 was achieved in the LOSO and the stratified 10-fold evaluation over the best method reproduced from previous works. For the tremor data, a difference of 0.01 in AUC is significant with *p*-value < 0.001, according to Hanley’s method [97]. Even with the increase in the AUC, the CNN multioutput (with FFT) evaluated through the stratified 10-fold evaluation does not seem to show a significant difference compared to the best reproduced method.

In the experiments performed in this study, the system proposed by Hssayeni et al. [15] (handcrafted features with Gradient Boost) presents a higher performance in terms of AUC among the shallow machine learning classifiers. This system shows a performance even higher (0.922) than the CNN single-output trained with raw signals. Additionally, the AdaBoost algorithm with FFT presents a good performance over the CNN single-output (but less than the multitask models). For this reason, these shallow approaches could be suitable for the development of systems dedicated only to the analysis of tremors or for systems based on multiple classifiers.

The remaining approaches for tremor detection based on shallow classification algorithms present lower performances, with sensitivities and specificities ranging from 80% to 84.0%. According to the results obtained in Table 5, the data representation based in MFCCs (AUC 0.905) could be considered a good trade between performance and computational burden due to the low number of features to be extracted.

In the related literature for tremor detection, sensitivities and specificities above 94% have been reported using configurations of four or six accelerometers, such as in the works of Rigas et al. [37], Keijsers et al. [33], Roy et al. [38] (with an additional EMG), and Salarian et al. [31] (with two gyroscopes). Although these methods show a great performance, the results obtained from the stratified 10-fold cross-validation using the multitask CNN presents similar performances, with AUC values up to 0.965, even considering the use of a single sensor.

These results suggest that it is feasible to develop optimized systems that can reduce the usability barriers such as the burden for the wearer when using multiple devices or the need for assistance for the correct placement of the sensors on the body.

### 4.5. Results of the Multitask CNN Per Patient

For further analysis, Table 7 shows the results per patient obtained from the multitask CNN models (FFT and triaxial raw data). The results presented are the mean values of six evaluations obtained per patient using LOSO validation, in a similar manner to the previous experiments.

Patients 4, 15, 17, and 18 did not preset tremors, therefore AUC values for tremor have not been obtained.

According to Table 7, the multitask CNN model with FFT presents values over the 0.935 for the AUC Context with a maximum value of 0.993, except for patient 12 with an AUC of 0.890. The AUC tremor present values over 0.900 in all cases, reaching a maximum of 0.981.

For the multitask CNN model trained with raw triaxial signals, the AUC context presents higher performances than the model trained with FFT. AUC Context values over 0.951 have been achieved with a maximum value of 0.998. In contrast, the AUC tremor presents a lower performance than the FFT, although this model presents AUC values over 0.902, patients 6, 11, and 12 present lower performances (between 0.826 and 0.887) reducing the overall performance of the model in tremor detection.

These results show an accurate performance in multiple task classification (context and tremor) in the majority of the patients in both models. The standard deviation of the six evaluations (trainings) present low variation for each patient and regardless of the classification task, which indicates that the model generalizes well with data it has not seen before using the configuration indicated in Section 3.5.3.

### 4.6. Comparison of the Performance and Number Trainable Parameters of the Deep Learning Models

Since the CNN models have presented higher performances for context and tremor detection, this section analyses the number of parameters that must be trained for each of the models. Table 8 shows a comparison of the number of trainable parameters and the performance of the CNN approaches for context and tremor detection.

According to Table 8, the best result for context detection was achieved using the multitask CNN with raw signal whereas the best result for tremor has been achieved by the multitask CNN with FFT, over the CNN single-output models.

On the one hand, the multitask CNN with FFT has 16,573 additional parameters compared with the single-output models. A reduced number of parameters could be used to implement an efficient system that avoids the execution of two models which requires a total of 240,442 parameters to perform the tremor assessment with a pipe such as the one shown in Figure 4.

On the other hand, the use of the multitask CNN with raw signals present a higher number of parameters (183,802 parameters). The use of this model could be justified by the fact that it presents the best performance for context detection and it does not require preprocessing, reducing the number of tasks that the system must perform to be used for long-term monitoring.

According to results achieved in the context and tremor classification, the subsequent experiments were performed with the CCN multioutput models to evaluate the methodology for tremor assessment.

### 4.7. Results of the Resting Tremor Assessment

Since the assessment of the symptom using amplitude and constancy is one of the main objectives of the proposed system, the results obtained from the complete system were compared with the clinical evaluations assessed by a neurologist from video recordings according to the UPDRS sections 3.17 and 3.18. The results for the amplitude and constancy were obtained using the best models (multitask CNN) trained during the LOSO evaluation of context and tremor classifiers.

#### 4.7.1. Results of the Amplitude Assessment

To evaluate the agreement between the acceleration level from the tremor band and the amplitude obtained through the clinical evaluation performed by the neurologist, a Kruskal–Wallis test was used, obtaining a chi-square = 20.84, *p* = 2.98 × 10^−5^ for the multitask CNN with FFT and a chi-square = 22.61, *p* = 1.23 × 10^−5^ for the multitask CNN with raw signals. Previously, a Shapiro–Wilk test was used to verify that the variables grouped according to the clinical score do not follow normal distributions.

**Figure 11:**
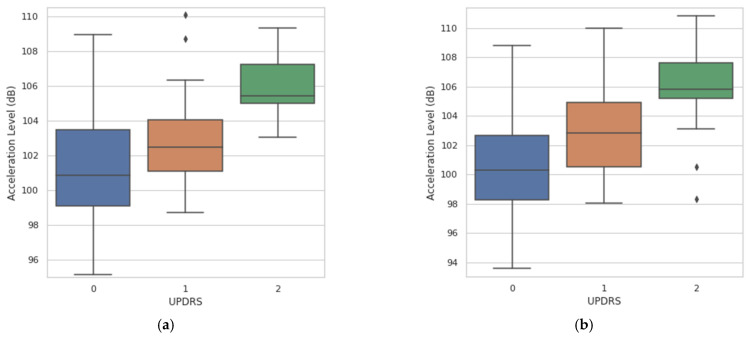
Agreement between the measurements of acceleration level derived from the resting tremor assessment and the UPDRS clinical score. (**a**) Agreement obtained with the multitask CNN with FFT. (**b**) Agreement obtained with the multitask CNN with raw signals.

Post-hoc Dunn’s test was used for pairwise comparisons. Both multitask CNN approaches were able to significantly differentiate (*p* ≤ 0.05) among all pairs of clinical scores (pairs adjacent and nonadjacent). Figure 11 shows the agreement between the acceleration level obtained from the multitask CNN models and the clinical evaluation (UPDRS 3.17).

#### 4.7.2. Results of the Constancy Assessment

For the tremor constancy evaluation, the correlation between the clinical evaluation and the constancy obtained by the proposed system for each of the weekly experimental tests was analyzed.

**Figure 12:**
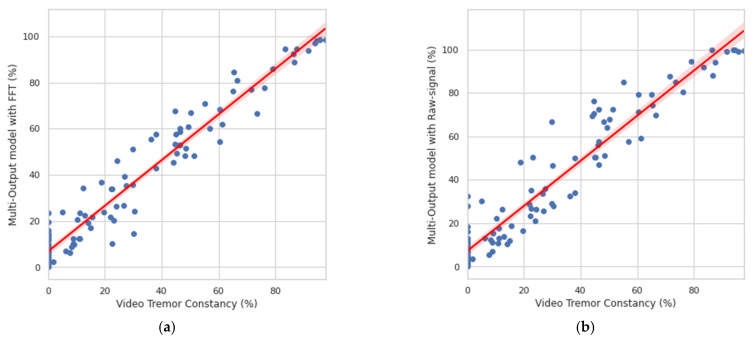
Correlation between the clinical evaluation and the constancy obtained by the system. (**a**) Correlation obtained with the multitask CNN with FFT. (**b**) Correlation obtained with the multitask CNN with raw signals.

As shown in Figure 12, a strong correlation was found between the constancy obtained by video evaluation and the multitask CNN approaches.

For the multitask CNN with FFT, a Person r = 0.969, *p* = 3.99 × 10^−64^ was found, while for the multitask CNN with raw signals, a Person r = 0.959, *p* = 1.56 × 10^−57^ was achieved. The results are shown in percentages to offer a better resolution for monitoring.

## 5. Discussion and Conclusions

This study analyzes the use of consumer smartwatches and their embedded accelerometers for monitoring and evaluating resting tremor in PD patients. The proposed method is composed of a context classifier that detects resting times, a tremor detector, and a module that assesses the amplitude and constancy of resting tremors. According to the results achieved, the proposed approaches based on CNN with multiple outputs are capable of deriving significant measures of the amplitude and constancy of resting tremors from the data collected during the performance of a set of programmed activities and rest periods.

The frequency response of the embedded accelerometer from the smartwatch has been verified to analyze the accuracy of the device for measuring the amplitude of the tremor. The results of the comparison with a reference accelerometer show a linear frequency response in the range of 3.15–20 Hz for amplitudes of 1 and 3 m/s^2^, and a linear frequency in the range of 5–20 Hz for an amplitude of 5 m/s^2^, which is a suitable frequency range for tremor analysis and activity detection. Additionally, the maximum amplitude range of the sensor (±2 g factory-set) has not been exceeded in the tests performed in this study for slight and mild tremor severities according to section 3.17 of the UPDRS guide.

It should be noted that an accurate selection of the data acquisition devices and sensors is relevant to develop systems with high performances. Optimized systems should be able to maintain high performance despite the variability of movement patterns or motor symptoms among patients, even by resorting to the use of a single sensor. In this study, the technical characteristics of a consumer smartwatch and its built-in accelerometer seem to be suitable for the acquisition of data to assess resting tremor in patients with PD.

The experimental protocol for data collection was carried out with 18 PD patients who performed a series of scripted activities alternated with rest periods. From the data collected in these experiments, several implementations of context classifiers (resting periods) and tremor classifiers have been evaluated.

For the implementation of the system, a hierarchical system was developed to simultaneously identify the resting times and the presence of tremors of the upper limbs. This approach opens opportunities for continuous monitoring in free-living environments without interrupting the patients’ daily lives. Additionally, with this approach, the sections identified as movements can be used to evaluate other PD motor symptoms such as bradykinesia.

When evaluating the complete system, the errors made by the context classifiers and the tremor detector did not appear to have a significant impact on the symptom assessment results; however, there was a risk of error propagation (if any of the classifiers present a high error). For this reason, novel algorithms, such as the proposed CNN classifiers (single and multioutput) shown in Section 3.5.1 and Section 3.5.3, could be used to improve the performance and reduce the risk of error propagation, even considering an increase in the number of parameters to be used for the classification models.

The evaluation of the context classifier using the LOSO methodology shows that the best results were obtained through the use of a multitask CNN, using the raw signals as input. Although this approach presents the best performance, the model has a higher number of trainable parameters (183,802) among the models presented in this work. The use of this model could be supported by the fact that the system implemented did not require preprocessing techniques besides the extraction of the acceleration level. The development of models similar to this could support the implementation of end-to-end systems where a complex feature extraction creation and evaluation process is not required.

For the tremor classifier, it was identified that the approach based on the multitask CNN with spectral representation presents a higher performance. As in [40], it was found that features extracted only from the frequency domain may allow tremor detection with sufficient accuracy to be used for the assessment of amplitude and constancy of tremor. This situation presents opportunities for the improvement and development of optimized systems for continuous operation, considering novel deep learning architectures and spectral data representations. For evaluating the performance of the resting tremor assessment, the present approach was compared with the clinical assessment made by the UPDRS sections 3.17 and 3.18. The results show a high agreement between the clinical assessment of the tremor amplitude and the acceleration level obtained from the data acquired by the smartwatch and using the multitask CNN models (Kruskal–Wallis chi-square = 20.84; *p* = 2.98 × 10^−5^ for the multitask CNN with FFT and a chi-square = 22.61; *p* = 1.23 × 10^−5^ for the multitask CNN with raw signals). The pairwise comparisons show a capacity to significantly differentiate between the absence of tremor (clinical score 0) and the presence of tremors with clinical scores of 1 and 2.

Additionally, a high correlation has been identified between the tremor constancy obtained through video evaluation and the constancy obtained from the multitask CNN (Person r = 0.969; *p* = 3.99 × 10^−64^ for the multitask CNN with FFT, and Person r = 0.959; *p* = 1.56 × 10^−57^ for the multitask CNN with raw signal).

The best results obtained in this study for multitask classification with LOSO evaluation show a sensitivity and specificity of 86.1% for tremor detection using the multitask CNN model with a spectral data representation while maintaining high performance in context detection (sensitivity of 89% and specificity of 89.1%).

These findings suggest that it is feasible to obtain significant measures of the amplitude and constancy with the accelerometers from consumer smartwatches and deep learning techniques based on multitask classification using convolutional networks to monitor patients in the early stages of the disease.

Although this paper presents an approach that analyzes tremor during rest periods based on a sequence of scripted activities (including exercises extracted from the UPDRS), the presented system is completely transferable for use in free-living environments, taking advantage of the capability of the context classifier to determine if the patient is at rest and is not performing any activity. During these periods, the whole system may be able to extract information that can be used to make an accurate analysis of the evolution of the symptom over time in an automated manner.

The smartwatch used in this study is capable of storing over 1000 discrete tests of six minutes (1.5 MB each test sampled at 50 Hz) in its internal free memory (2 GB). A high amount of data storage may be required to store the accelerometer data for continuous analysis—for example, performed over several months.

A possible solution to handle a high amount of data is the implementation of on-device processing (edge computing) to reduce the amount of data stored in the device. Additionally, it is possible to use cloud storage, for both inertial signals and the tremor assessment results obtained from automatic identification systems such as the one proposed in this work. These solutions can be implemented by taking advantage of the processing and wireless communication capabilities of the smart wearable devices [64].

The main contributions of this paper are the characterization of the embedded accelerometer of a consumer smartwatch to identify its accuracy to measure the amplitude of the tremor to be used as an indicator of the severity of the symptom, using standardized metrics (acceleration level) and reference values for measuring vibration levels. Additionally, the implementation and evaluation of novel approaches based on multitask CNNs to assess the resting tremor in PD. These approaches have been evaluated using both raw signals and spectral data representations as inputs. The results obtained from these models indicate an increase in the performance for context and tremor detection when compared with the reproduction of systems proposed in the related literature.

Despite the potential advantages of the system, it should be noted that an accurate amplitude evaluation requires a properly calibrated sensor to provide an accurate representation of the movement. Additionally, it must be taken into account that there is a possibility that the system may underestimate the highest amplitude scores due to the configuration of the analysis range established at the factory in the sensors (±2 g). Future studies will analyze the influence of the sensor for the evaluation of patients in more advanced stages of the disease, who could present tremors with higher amplitudes.

Although certain technical limitations in the smartwatches were identified, the results suggest that the approach based on consumer smartwatches and deep learning techniques can be feasible and derive significant measures for symptom monitoring in patients in the early stages of the disease. The use of affordable wearable technology shows high potential and opportunities for the development of tools that can be integrated into the routine care and assessment of PD patients. In this way, affordable tools can be developed to improve the management and monitoring protocol through objective evaluations, which can be made automatically and in a continuous manner, enabling the development of effective treatments in clinical and ambulatory settings.

## Figures and Tables

**Table 1:** Summary of highlighted research articles for tremor analysis using wearable sensors.

Work	Year	Sensors and Location	Participants	Analysis Methods	Main Aims or Findings	Main Results
Patel et al. [36]	2010	8 uniaxial accelerometers, located on the arms and legs	12 PD patients	Machine learning: Support Vector Machines	The results indicate that is possible to estimate clinical scores with a low error	Error values of 3.4% for tremor detection using hand-crafted features.
Rigas et al. [37]	2012	6 accelerometers located on the wrists, ankles, sternum, and the waist	23 subjects (18 PD patients and 5 healthy controls)	Hidden Markov model	High accuracy in tremor detection. It is possible to discriminate tremors from other PD symptoms.	Accuracy of 87% for tremor severity, maximum specificity 97%, and sensitivity 95%
Roy et al. [38]	2013	EMG and 4 accelerometers located on both forearms and shanks	11 PD for training, and 12 for testing (8 PD + 4 healthy controls)	Machine learning: Dynamic neural network	The use of a hybrid sensor and a neural network to detect tremor during unconstrained activities	Overall mean specificity of 90.2% and sensitivity of 92.9% for tremor detection
Tzallas et al. [39]	2014	4 triaxial accelerometers and 1 gyroscope on both wrists, ankles, and the waist	20 PD patients for short-term analysis and 24 for long-term analysis	Machine learning	The authors propose a system for continuous evaluation of tremor and motor symptoms	Accuracy of 87% for tremor classification
Ahlrichs et al. [40]	2014	Triaxial accelerometer located on the wrist	76 subjects (64 PD for testing and 12 PD for training)	Machine learning: Support vector machines	The results indicate that frequency domain features may be enough to detect tremor	Specificity of 88.4% and sensitivity of 89.4%
Kostikis et al. [57]	2015	Smartphone accelerometer and gyroscope, located on the wrists with a glove	25 PD patients and 20 age-matched healthy controls	Machine learning	The authors propose the use of consumer smartphones to assess tremor	Accuracy of 82% for PD patients and 90% for healthy controls
Braybrook et al. [43]	2016	A triaxial accelerometer located on the wrist of the most affected side	85 PD patients	Threshold method using a spectral analysis	The authors propose a system for ambulatory assessment of PD tremor	Specificity of 92.5% and selectivity of 92.9% for tremor detection
García-Magariño et al. [45]	2016	Smartphone with triaxial accelerometer used in unconstrained environments	11 PD with tremors 10 subjects without tremor	Algorithmic approach	The authors propose a smartphone-based application for detecting hand tremor	Specificity of 95.8% and Sensitivity of 99.5% for tremor detection
Jeon et al. [46]	2017	A watch-like device with an accelerometer and gyroscope located on the wrist and finger of both hands	85 PD patients	Machine learning: Several algorithms	The authors show an accurate scoring system for estimating the tremor severity	Accuracy of 85.5%, and an RMSE of 0.410 (on five classes according to UPDRS)
Pulliam et al. [47]	2018	Triaxial accelerometer and gyroscope located on both wrists and ankles	13 PD patients	Regression models	The use of wearable sensors to quantify the dose–response for several symptoms	AUC 0.89 for tremor detection.
Kim et al. [50]	2018	Triaxial accelerometer and gyroscope located on the wrist and finger of both hands	92 PD patients	Deep learning: Convolutional neural networks (CNNs)	The use of CNN outperforms machine learning approaches	Accuracy of 0.85, and RMSE of 0.35
López-Blanco et al. [51]	2019	Consumer smartwatch (accelerometer and gyroscope)	22 PD patients	The tremor intensity calculated through RMS of the gyroscope signal	The use of consumer smartwatches for tremor quantification is reliable and well-correlated with clinical scores.	Spearman coefficient between UPDRS scores and smartwatch measurements for the intensity of 0.81 (*p* < 0.001)
Hssayeni et al. [15]	2019	Triaxial gyroscope located on the wrist and ankle of the most-affected side	24 PD patients	Machine and deep learning: Recurrent neural networks	The authors propose the use of gradient tree boosting and Long short-term memory networks	A correlation r = 0.93 with LOSO using gradient tree boosting algorithm
Pierleoni et al. [52]	2019	Watch like device with a triaxial accelerometer, gyroscope, and magnetometer	40 PD patients	Threshold method	A method for continuous and real-time monitoring of PD using wearables and Cloud services	Accuracy of 97.7% for tremor detection
Battista et al. [53]	2020	Watch-like device with a triaxial accelerometer	20 PD patients	Threshold method	A device for continuous monitoring of PD tremor, increasing the discrimination with normal daily activities	Linear correlation with the UPDRS constancy r = 0.744 (*p* = 0.0004)
Van Brummelen et al. [54]	2020	Consumer devices with triaxial accelerometers	10 patients with PD and 10 with essential tremor	Spectral analysis	The authors evaluate the performance of the accelerometers of consumer devices.	Consumer devices could be suitable to analyze the peak frequency in PD tremor
Mahadevan et al. [55]	2020	Watch-like IMU (results reported with the use of the accelerometer)	31 PD and 50 healthy controls for evaluating the tremor detector	Threshold method and machine learning	Sensor measures of resting tremor present high agreement with clinical scoring	Tremor classifier accuracy of 83%. Pearson correlation of 0.97 for tremor constancy
San Segundo et al. [56]	2020	Wrist-worn triaxial accelerometers	12 patients with PD	Deep learning: Convolutional neural networks	Evaluation of novel preprocessing methods and algorithms in free-living and laboratory settings	Error lower than 5% when estimating the percentage of tremor in a laboratory setting

**Table 2:** Percentage of deviation of the smartwatch compared to the reference accelerometer.

	Frequency (Hz)								
Amplitude	3.15	4	5	6.3	8	10	12.5	16	20
5 m/s^2^	-	-	1.33%	1.47%	1.44%	0.67%	0.03%	−2.38%	−4.79%
3 m/s^2^	−0.82%	−0.51%	1.63%	1.80%	1.85%	0.79%	−0.27%	−1.94%	−4.29%
1 m/s^2^	−4.65%	−0.3%	0.27%	−0.31%	0.47%	0.0%	0.69%	−2.01%	−4.89%

**Table 3:** Summary of the data representations used in the tremor detector.

Data Representation	Number of Features	Description of the Features
FFT	64	Symmetric part of FFT obtained from the Euclidean Norm.
MFCCs [49,56]	36 (12 × 3 channels)	Mel frequency cepstral coefficients adapted toinertial signals.
Hssayeni et al. [15]	39	Power in bands (4–6 Hz and 0.5–15 Hz), autocorrelation features, spectral entropy, first and second dominant frequencies and magnitudes, cross-correlations between pairs of the axis.
Mahadevan et al. [55]	64 (8 × 8 preprocessed signals)	RMS, signal range, signal entropy, dominant frequency and magnitude, the ratio of the dominant frequency band to total energy, spectral flatness, spectral entropy.
Raw signal	384 (128 × 3 channels)	The raw triaxial signal obtained for the accelerometer.

**Table 4:** Results for the leave-one-subject-out (LOSO) evaluation of the context classifier using different approaches.

Data Representation	# of Features	Classifier	Sensitivity	Specificity	AUC
HAR [77] (baseline)	290	AdaBoost	88.5% (5.56)	88.6% (5.58)	0.953 (0.037)
FFT	64	AdaBoost	88.6% (4.88)	88.6% (4.89)	0.954 (0.029)
FFT	64	Gradient Boost	89.8% (4.82)	89.8% (4.82)	0.961 (0.029)
Raw signal	384	CNN (with GAP)	89.8% (5.00)	89.8% (5.01)	0.962 (0.029)
Raw signal	384	multitask CNN	92.9% (3.42) ^1^	93.0% (3.58) ^1^	0.981 (0.017) ^1^
FFT	64	multitask CNN	89.0% (4.91) ^1^	89.1% (4.90) ^1^	0.960 (0.025) ^1^

^1^ Values obtained only from the context classifier output.

**Table 5:** Results of the LOSO evaluation for different classifiers with data of patients with tremor.

Data Representation	# of Features	Classifier	Sensitivity	Specificity	AUC
FFT (baseline)	64	SVM	81.1% (5.89)	80.5% (6.67)	0.872 (0.045)
FFT	64	AdaBoost (100 estimators)	84.3% (3.20)	84.4% (3.42)	0.918 (0.025)
MFCCs [49]	36	AdaBoost (100 estimators)	82.6% (4.86)	82.2% (4.65)	0.905 (0.040)
Mahadevan et al. [55]	64	Random Forest (10 estimators)	82.3% (5.88)	84.0% (4.51)	0.893 (0.049)
Hssayeni et al. [15]	39	Gradient Boost (170 estimators)	84.1% (4.52)	84.0% (4.13)	0.922 (0.035)
Raw signal	384	CNN (with GAP)	84.1% (4.30)	84.0% (4.20)	0.915 (0.043)
Raw signal	384	multitask CNN	85.0% (5.15) ^2^	85.3% (5.13) ^2^	0.923 (0.039) ^2^
FFT	64	multitask CNN	86.1% (5.37) ^2^	86.1% (5.49) ^2^	0.936 (0.024) ^2^

^2^ Values obtained only from the tremor detector output.

**Table 6:** Comparison of the performance of the best systems reproduced from previous works and the best CNN multitask systems.

System	Classification Task	LOSO AUC	Stratified 10-Fold AUC
HAR features [77] + Adaboost (with 100 estimators)	Context	0.962 (0.029)	0.970 (0.003)
multitask CNN with raw signal (ours)	Context	0.981 (0.017) ^3^	0.990 (0.002) ^3^
Hssayeni et al. [15] (Specific tremor features)	Tremor	0.922 (0.035)	0.956 (0.003)
multitask CNN with FFT (ours)	Tremor	0.936 (0.024) ^4^	0.965 (0.003) ^4^

^3^ Value obtained only from the context classifier output. ^4^ Value obtained only from the tremor detector output.

**Table 7:** Area under the curve (AUC) results per patient of the multitask CNN models using LOSO evaluation.

Patient ID	Multitask CNN with FFT		Multitask CNN with Raw Signals	
	AUC Context	AUC Tremor	AUC Context	AUC Tremor
1	0.961 (0.003)	0.962 (0.004)	0.969 (0.005)	0.948 (0.010)
2	0.980 (0.002)	0.960 (0.002)	0.994 (0.0002)	0.926 (0.002)
3	0.960 (0.001)	0.981 (0.001)	0.975 (0.006)	0.978 (0.002)
4	0.956 (0.006)	-	0.972 (0.001)	-
5	0.950 (0.001)	0.928 (0.003)	0.971 (0.001)	0.902 (0.004)
6	0.935 (0.002)	0.913 (0.002)	0.952 (0.001)	0.848 (0.002)
7	0.936 (0.001)	0.933 (0.002)	0.981 (0.0002)	0.952 (0.002)
8	0.940 (0.003)	0.933 (0.004)	0.982 (0.003)	0.959 (0.005)
9	0.949 (0.003)	0.959 (0.005)	0.976 (0.002)	0.954 (0.005)
10	0.962 (0.003)	0.900 (0.003)	0.995 (0.0004)	0.904 (0.003)
11	0.975 (0.003)	0.915 (0.005)	0.990 (0.001)	0.826 (0.010)
12	0.890 (0.003)	0.924 (0.004)	0.951 (0.005)	0.887 (0.003)
13	0.985 (0.003)	0.909 (0.003)	0.995 (0.0003)	0.927 (0.003)
14	0.982 (0.0003)	0.932 (0.001)	0.996 (0.0005)	0.930 (0.007)
15	0.984 (0.001)	-	0.997 (0.0003)	-
16	0.957 (0.003)	0.959 (0.002)	0.969 (0.005)	0.935 (0.002)
17	0.981 (0.0001)	-	0.991 (0.0004)	-
18	0.993 (0.001)	-	0.998 (0.0004)	-

**Table 8:** Comparison of the number of trainable parameters and the performance of the convolutional models.

Model	Data Representation	Number of Trainable Parameters	AUC Context	AUC Tremor
CNN context (single output)	Raw signal	120,221	0.962 (0.029)	-
CNN tremor (single output)	Raw signal	120,221	-	0.915 (0.043)
multitask CNN	Raw signal	183,802	0.981 (0.017)	0.920 (0.044)
multitask CNN	FFT	136,794	0.960 (0.025)	0.936 (0.024)

## Data Availability

The data presented in this study are available on request from the corresponding author. The data are not publicly available because they contain protected patient health information.
